# Feeder-supported in vitro exercise model using human satellite cells from patients with sporadic inclusion body myositis

**DOI:** 10.1038/s41598-022-05029-w

**Published:** 2022-01-20

**Authors:** Yuqing Li, Weijian Chen, Kazumi Ogawa, Masashi Koide, Tadahisa Takahashi, Yoshihiro Hagiwara, Eiji Itoi, Toshimi Aizawa, Masahiro Tsuchiya, Rumiko Izumi, Naoki Suzuki, Masashi Aoki, Makoto Kanzaki

**Affiliations:** 1 Department of Orthopaedic Surgery, Graduate School of Medicine, Tohoku University, Sendai, Japan; 2 Graduate School of Biomedical Engineering, Tohoku University, 6-6-04-110, Aramaki, Aoba-ku, Sendai, 980-8579 Japan; 3 Department of Orthopaedic Surgery, Tohoku Rosai Hospital, Sendai, Japan; 4 Department of Nursing, Tohoku Fukushi University, Sendai, Japan; 5 Department of Neurology, Tohoku University Graduate School of Medicine, Sendai, Japan

**Keywords:** Biological techniques, Biological models, Cell biology, Mechanisms of disease, Diseases, Neurological disorders, Movement disorders, Neuromuscular disease

## Abstract

Contractile activity is a fundamental property of skeletal muscles. We describe the establishment of a “feeder-supported in vitro exercise model” using human-origin primary satellite cells, allowing highly-developed contractile myotubes to readily be generated by applying electrical pulse stimulation (EPS). The use of murine fibroblasts as the feeder cells allows biological responses to EPS in contractile human myotubes to be selectively evaluated with species-specific analyses such as RT-PCR. We successfully applied this feeder-supported co-culture system to myotubes derived from primary satellite cells obtained from sporadic inclusion body myositis (sIBM) patients who are incapable of strenuous exercise testing. Our results demonstrated that sIBM myotubes possess essentially normal muscle functions, including contractility development, de novo sarcomere formation, and contraction-dependent myokine upregulation, upon EPS treatment. However, we found that some of sIBM myotubes, but not healthy control myotubes, often exhibit abnormal cytoplasmic TDP-43 accumulation upon EPS-evoked contraction, suggesting potential pathogenic involvement of the contraction-inducible TDP-43 distribution peculiar to sIBM. Thus, our “feeder-supported in vitro exercise model” enables us to obtain contractile human-origin myotubes, potentially utilizable for evaluating exercise-dependent intrinsic and pathogenic properties of patient muscle cells. Our approach, using feeder layers, further expands the usefulness of the “in vitro exercise model”.

## Introduction

Myotubes obtained under conventional culture conditions normally exhibit little or no contractile activity due to a lack of excitation stimuli from motor neurons. Recently, “in vitro exercise models” with contractile myotubes generated by applying electric pulse stimulation (EPS) in culture^1,2^ have been widely utilized for investigating the impacts of actual contractile activity on muscle cell properties including exercise-inducible myokine secretion^3^, improved insulin sensitivity^4,5^, and sarcomere formation^6^. However, this widely used model has limitations, especially as regards human myotubes derived from primary satellite cells. For example, given that human myotubes generally display a very flat and spread-out morphology, attaching firmly to the culture substratum, human myotubes usually have much poorer contractile activity than murine myotubes originating from the C2C12 muscle cell line under conventional culture conditions even with EPS treatment^7,8^. Moreover, while inoculation with a very high cell-density of human-origin myoblasts (e.g. more than five-fold higher than murine C2C12 cells) slightly improves their contractility development and myokine upregulations upon EPS^7^, it is usually difficult to obtain sufficient numbers of human satellite cells from biopsy samples, particularly from those of patients suffering from rare muscle diseases such as sporadic inclusion body myositis (sIBM)^9^. Thus, a useful and simple method allowing the generation of human myotubes capable of vigorously contracting in the culture system, which would markedly boost not only basic muscle research but also the diagnostic use of muscle cells obtained from patient biopsy samples, a very limited resource, is eagerly anticipated.

sIBM is an acquired progressive muscle disease that typically affects patients older than 50 years of age^10,11^ and is pathologically characterized by sarcoplasmic rimmed vacuole formation along with protein aggregates of p62^12^ and mis-localized cytoplasmic TAR DNA-binding protein of 43 kDa (TDP-43)^13^ as well as mitochondrial abnormalities in affected muscle fibers^14,15^. This disease results in slowly progressive and often asymmetric weakness with finger flexors and knee extensors being predominantly affected^16^. To date, the basic cellular functions of muscles from sIBM patients have not been well investigated, though one study demonstrated that primary sIBM myoblasts show no apparent morphological abnormalities and are fully capable of forming normal myotubes that can be properly innervated with rat embryo spinal cord in an in vitro co-culture system^17^. Since the outcome of this degenerative muscle disease is progressive loss of muscle contractile activity, analysis of sIBM muscle cells, ideally under contractile (exercising) conditions, is necessary for gaining meaningful insights into the pathogenic mechanisms underlying this disease.

We herein succeeded in establishing a new “in vitro exercise model” applicable to human-origin muscle cells, which is based on a co-culture system with species-different (murine) fibroblasts as feeder cells efficiently supporting the contractility development of human myotubes upon appropriate EPS treatment. We applied this “feeder-supported in vitro exercise model” to sIBM myotubes derived from the primary satellite cells of sIBM patients and examined their functional muscle properties in response to EPS-evoked contraction by comparing them with those of myotubes derived from healthy human subjects. Our results indicate sIBM myotubes to have basically the same muscular properties upon EPS-evoked contraction as normal myotubes, though some of sIBM myotubes often exhibited cytoplasmic TDP-43 accumulations in response to EPS-evoked contraction, which was not detected in healthy controls. While such an exercise-loading test would not be feasible for patients suffering from severe diseases of muscular dysfunction, such as sIBM, the culture system developed in this study should allow us to better understand a patient’s muscle cell condition, particularly responses to actual contraction loading, potentially providing important diagnostic information allowing for customized therapies.

## Results

### Feeder cells support development of EPS-inducible contractile activity and de novo sarcomere formation in human myotubes

**Figure 1:**
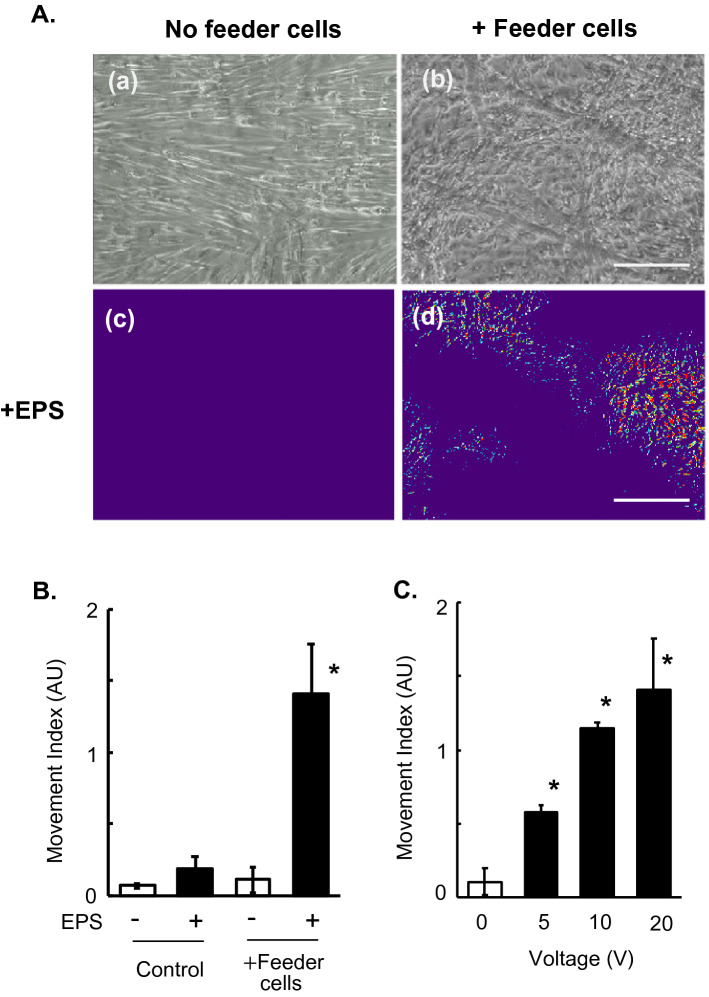
Contractile activity of human myotubes with or without mouse fibroblast feeders. After 7–8 days of differentiation, EPS (1 Hz frequency, 4-ms duration, 20 V/25 mm) was applied to differentiated human myotubes (derived from HSMM) cultured without (*panels a and c*) or with (*panels b and d*) feeder 3T3L1 fibroblasts for a total of 24 h (8 h × 3 times with 1 h interval between sessions). Images of myotubes were then taken during the last 15 min of the total EPS session (Supplementary Movies S1 and S2). (**A**) The pseudo-colored differential images (*panels c and d*), reflecting contractile area and ability, were produced as described in the “Methods” section. The bright-field images of the same area (*panels a and b*) are also presented. Scale bar = 250 μm. Three independent experiments were performed and representative images are presented. (**B**) The Movement Index was determined as described in the “Methods” section. *Significant effects compared to No-EPS (*n* = 3, *p < 0.05). (**C**) The EPS intensity-dependent contractile activity of the feeder-supported in vitro exercise model was determined by the Movement Index at varying voltages (5, 10 and 20 V/25 mm) of EPS (1 Hz frequency, 4-ms duration). The statistical significance was determined by ANOVA with Tukey’s multiple comparison test. *Significant effects compared to No-EPS (0 V EPS) (*n* = 3, *p < 0.05).

As we previously reported^7^, myotubes derived from human skeletal muscle myoblasts (HSMM) displayed a flattened myotubular morphology reflecting their firm adhesion to the substratum. In the absence of 3T3L1 feeder cells, though human myotubes appeared morphologically well-differentiated, they exhibited minimal contraction in response to EPS (Fig. 1) even after applying the same EPS treatment (1 Hz, 4-ms, 20 V/25 mm) that would have effectively endowed vigorous contractile ability in murine C2C12 myotubes^2^ and in hybrid myotubes generated from murine C2C12 and human muscle cells^7^. In contrast, human myotubes differentiated on the feeder cells exhibited round cylinder-like myotubular morphology embedded within surrounding feeders and displayed obvious contractile activity in response to the EPS treatments (1 Hz, 4-ms, 20 V/25 mm for total 24 h). Their contraction activity, as assessed by the movement index during the final ~ 15 min. of the EPS treatment, was dependent on an EPS intensity of at least until 20 V (Fig. 1C). In the absence of EPS treatment, no spontaneous contraction was observed in human myotubes even on the feeder layer of fibroblasts.

**Figure 2:**
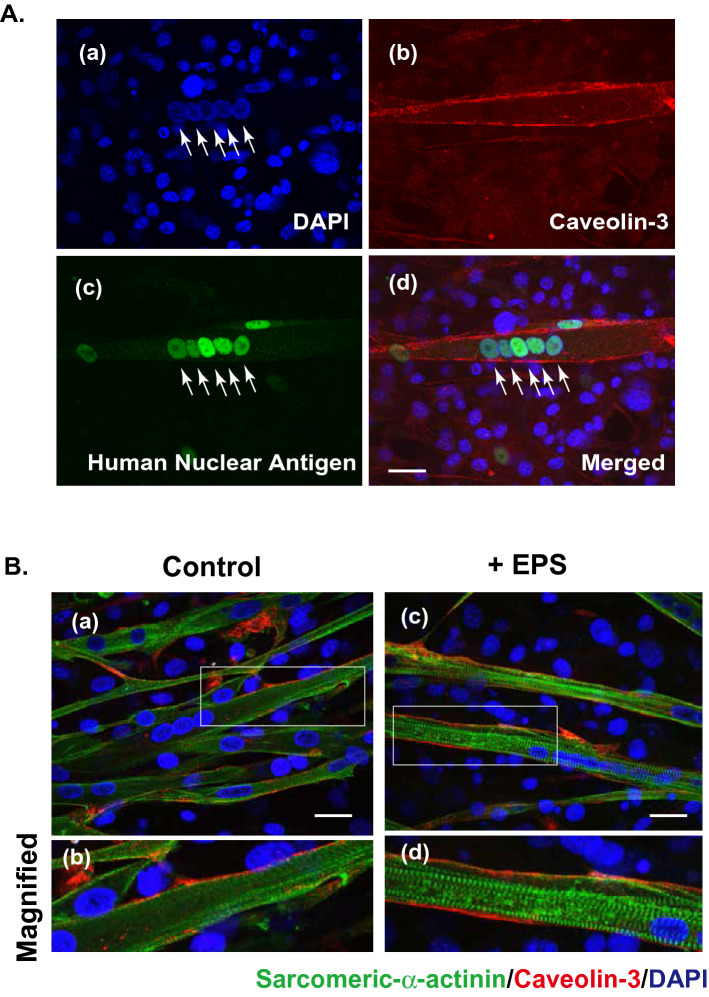
Sarcomere formation in contracting human myotubes on mouse fibroblast feeders. (**A**) After 7–8 days of differentiation, the differentiated human myotubes (derived from HSMM) on the feeder layers of mouse 3T3L1 fibroblasts were fixed and then observed for myotubular formation by using anti-human nuclear antigen (anti-HNA) and anti-Caveolin 3 antibodies, as described in the “Methods” section. DAPI was used for nuclear staining. Scale bar = 25 μm. Three independent experiments were performed, and representative images are presented. (**B**) The differentiated human myotubes on the mouse fibroblast feeders were subjected to either no (*panels a and b*) EPS or (*panels c and d*) EPS treatment (1 Hz frequency, 4-ms duration, 20 V/25 mm for a total 24 h of with intermittent intervals) and then fixed for evaluating sarcomere formation status by using anti-sarcomeric-α-actinin (*green*) and anti-Caveolin 3 (*red*) antibodies. DAPI was used for nuclear staining (*blue*). Scale bar = 25 μm. Magnified images (*panels b and d*) of the white boxes in *panels a and c*, respectively, are also presented. Three independent experiments were performed and representative images are presented.

Immunofluorescent staining using anti-human nuclear antigen (HNA) confirmed that nuclei of the myotubes were all positive for anti-HNA in the co-presence of murine 3T3L1 fibroblast feeders in the same culture (Fig. 2A). This result implies that human myoblasts (activated satellite cells) do not fuse with murine fibroblasts, though human myoblasts and murine C2C12 myoblasts can fuse with each other to form a hybrid myotube^7^. As expected from their contractile activity (Fig. 1), sarcomere structure, as assessed by sarcomeric α-actinin staining, was frequently observed in the myotubes grown on the feeder cells subsequent to EPS treatment (Fig. 2B).

Taking these data together, we can reasonably assume that although human myotubes alone without feeders exhibited poor or little contractile activity even when subjected to EPS treatment, feeder layers of fibroblasts provide a favorable substratum for human myotubes, resulting in better development of their contractile activity and de novo sarcomere structure formation upon EPS treatment.

### Myotubes arising from satellite cells obtained from sIBM patients similarly exhibit vigorous contractile activity along with CXCL1 upregulation upon EPS treatment

**Figure 3:**
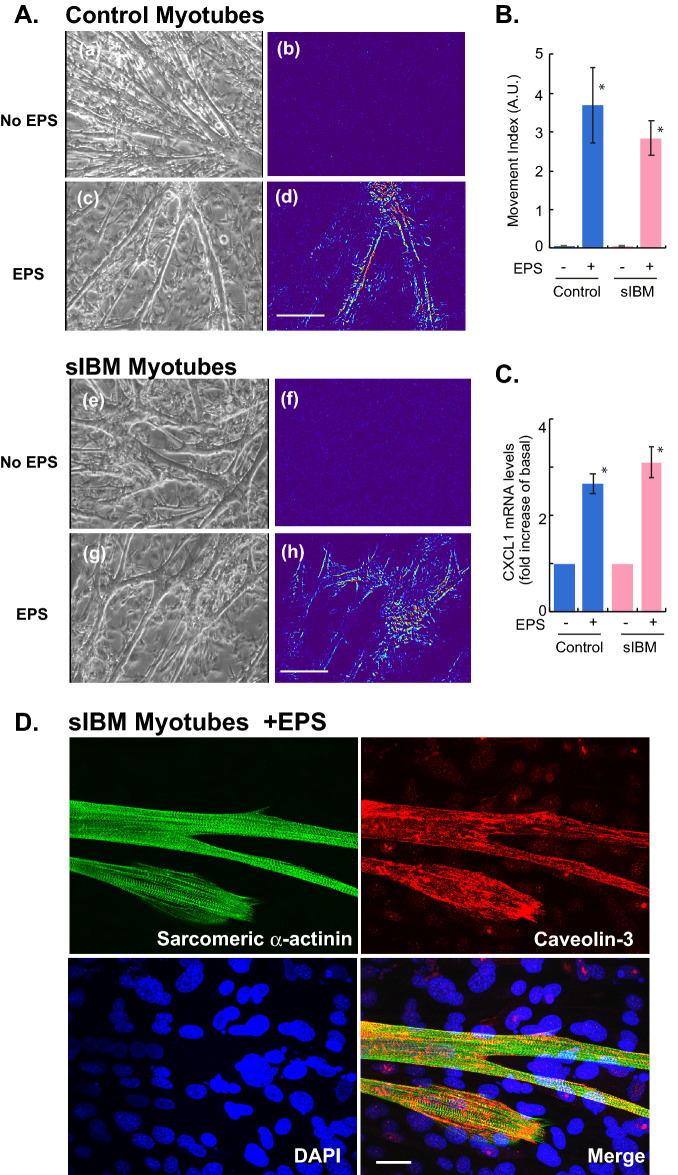
Contractile activity and myokine upregulation in myotubes derived from primary satellite cells of sIBM patients and of healthy subjects. (**A**) By employing the feeder-supported in vitro exercise model, the differentiated myotubes originating from primary satellite cells obtained from biopsy samples of healthy control subjects and sIBM patients were treated with or without EPS at 20 V/25 mm, 1 Hz, 4-ms duration for total of 24 h and their contractility in response to EPS was examined. Images of myotubes were taken during the last 15 min. of the total EPS session. The pseudo-colored differential images (*panels b, d, f and h*), reflecting contractile area and ability, were produced as described in the “Methods” section. The bright-field images of the same area (*panels a, c, e and g*) are also presented. Scale bar = 250 μm. (**B**) The Movement Index was determined as described in the “Methods” section. *Significant effects compared to No-EPS (*n* = 3, *p < 0.05). (**C**) Total RNA was extracted and mRNAs for human CXCL1 and RPLP0 were evaluated by real-time PCR analysis. Data were normalized using human RPLP0 transcripts and the values are presented as fold increases as compared with basal (No-EPS treatment) (*n* = 3, *p < 0.05). (**D**) After EPS treatment, the sIBM myotubes were fixed for evaluating sarcomere formation status by using anti-sarcomeric-α-actinin (*green*) and anti-Caveolin 3 (*red*) antibodies. DAPI was used for nuclear staining (*blue*). Scale bar = 25 μm. Three independent experiments were performed and representative images are presented.

Taking advantage of the in vitro exercise model co-cultured with feeder fibroblasts (feeder-supported in vitro exercise model), we next examined the contractile activity of myotubes derived from primary satellite cells obtained from biopsy samples of healthy subjects or sIBM patients. As shown in Fig. 3, myotube formation of sIBM satellite cells (*panels e–h*) on feeder cells was equivalent to that of control satellite cells (*panels a–d*), but no spontaneous contractile activity was detected in either sIBM (*panels e* and *f*) or control myotubes (*panels a and b*) when EPS treatment was lacking. However, EPS treatment efficiently endowed vigorous contractile ability in both control (*panels c* and *d*) (Supplementary Movie 1) and sIBM myotubes (*panels g* and *h*) (Supplementary Movie 2), and their EPS-evoked contractions were similar, according to their movement index results (Fig. 3B). Sarcomere structures were also observed in sIBM myotubes (Fig. 3D) after EPS treatment, similar to the observations in control myotubes (Fig. 2B, *panels c* and *d*). These results indicate that the sIBM myotubes fully retained EPS-dependent de novo sarcomere formation and the consequent vigorous contractile activity lasted for at least the duration of the 24-h EPS treatment.

CXCL1 has been recognized as a myokine that is upregulated in response to muscle contractile activity in vivo^2^ and in vitro^4,7^. We therefore examined CXCL1 mRNA expression levels in human myotubes of the feeder-supported in vitro exercise model (Fig. 3C). Species-specific RT-PCR analysis^7^ clearly demonstrated that EPS-evoked contractile activity significantly increases CXCL1 mRNA expression levels in both sIBM myotubes and healthy control myotubes. The mRNA levels of IL-6, another well-established myokine^3,18^, were also similarly upregulated by EPS treatment in both myotubes (*data not shown*). Hence, consistent with the movement index results, sIBM myotubes on the feeders have similar responsiveness to EPS-evoked contraction in terms of contraction-inducible CXCL1 upregulation.

### sIBM Myotubes exhibit cytoplasmic TDP-43 accumulation after EPS-evoked contraction

**Figure 4:**
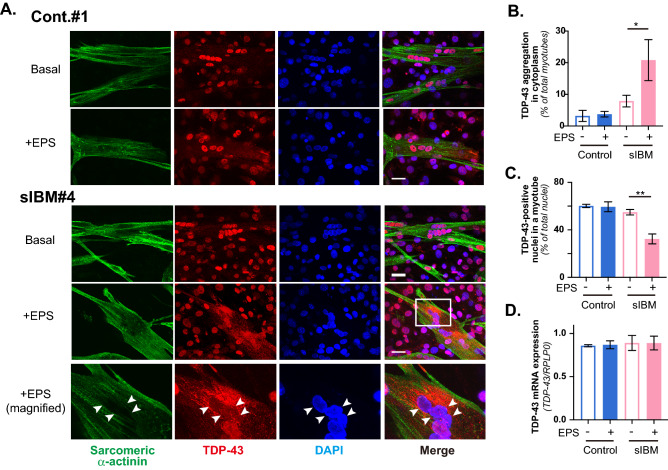
Subcellular localization of TDP-43 in sIBM myotubes of feeder-supported in vitro exercise model. (**A**) Six human myotube preparations originating from 3 healthy control subjects (Cont. #1–#3) and 3 sIBM patients (sIBM#4–#6) were subjected to immunofluorescent staining analysis. After 7–8 days of differentiation, the differentiated human myotubes on the feeder layers of mouse 3T3L1 fibroblasts were subjected to EPS treatments, fixed and then observed for subcellular localization of TDP-43 by using anti-TDP-43 and anti-sarcomeric-α-actinin antibodies, as described in the “Methods” section. DAPI was used for nuclear staining. Scale bar = 25 μm. Three independent experiments were performed, and one healthy control and one sIBM myotube are each presented as the representative images. Note that cytoplasmic TDP-43 accumulation is observed in sIBM myotubes after EPS-evoked contraction (*lower two row panels*). A magnified image of the white box (sIBM myotubes + EPS) is also presented (*the bottom panels*). Scale bar = 25 μm. (**B,C**) For quantification, 5 different fields in the images (of which each field usually contained 6–12 myotubes) were taken and the numbers of myotubes possessing intracellular TDP-43 accumulations as well as the numbers of TDP-43-positive nuclei in the myotube were counted, as described in the “Methods” section. Three independent experiments using the same cell stock samples were performed. Quantification of the ratio of myotubes displaying cytoplasmic TDP-43 accumulations (**B**) and the ratio of TDP-43-positive nuclei in the myotube (**C**) were determined from more than 60 myotubes in three independent experiments. The data are shown as the mean ± SE (healthy control and sIBM myotubes, *n* = 3 each). The statistical significance of differences was analyzed using ANOVA with Dunnett’s multiple comparison test; *p < 0.05 and **p < 0.01 indicating the effects of EPS treatment. (**D**) Total RNA was extracted and mRNAs for human TDP-43 and RPLP0 were evaluated by real-time PCR analysis. Data were normalized using human RPLP0 transcripts. This graph shows the results of three independent experiments performed using the same cell stock samples (healthy control and sIBM myotubes, *n* = 3 each).

**Figure 5:**
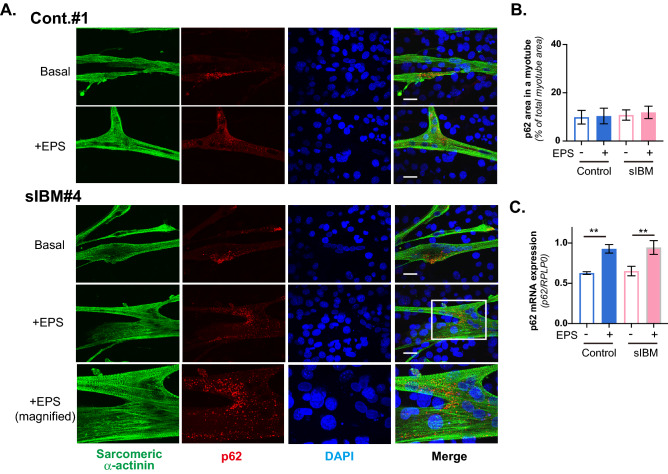
Subcellular localization of p62 in myotubes of feeder-supported in vitro exercise model. (**A**) Six human myotube preparations originating from 3 healthy control subjects (Cont. #1–#3) and 3 sIBM patients (sIBM#4–#6) were subjected to immunofluorescent staining analysis. After 7–8 days of differentiation, the differentiated human myotubes on the feeder layers of mouse 3T3L1 fibroblasts were subjected to EPS treatments, fixed and then observed for subcellular localization of p62 by using anti-p62 and anti-sarcomeric-α-actinin antibodies, as described in the “Methods” section. DAPI was used for nuclear staining. Scale bar = 25 μm. Three independent experiments were performed, and one healthy control and one sIBM myotube are each presented are presented as the representative images. A magnified image of the white box (sIBM myotubes + EPS) is also presented (*the bottom panels*). Scale bar = 25 μm. (**B**) For quantification, 5 different fields in the images (of which each field usually contained 6–12 myotubes) were taken and the p62 puncta area to the total area of a myotube was calculated, as described in the “Methods” section. Quantification of the ratio of the p62 area to the total myotube area was determined from more than 60 myotubes in three independent experiments using the same cell stock samples. The statistical significance of differences was analyzed using ANOVA with Dunnett’s multiple comparison test. (**C**) Total RNA was extracted and mRNAs for human p62 and RPLP0 were evaluated by real-time PCR analysis. Data were normalized using human RPLP0 transcripts. The graph shows the results of three independent experiments using the same cell stock samples (healthy control and sIBM myotubes, *n* = 3 each, **p < 0.01).

Pathological deposits of several proteins such as TDP-43 and p62 have been observed in muscles of sIBM patients^19,20^, and immunostaining analysis using anti-phospho-TDP-43 and anti-p62 antibodies confirmed that biopsy samples of all three of our sIBM patients possessed myofibers containing pTDP-43-positive and p62-positive granular structures (Supplementary Information, Fig. S1). We therefore performed immunofluorescent staining analysis of both TDP-43 (Fig. 4) and p62 (Fig. 5) in sIBM myotubes with or without EPS-evoked contraction by employing the feeder-supported in vitro exercise model. In healthy control myotubes, TDP-43 was mainly observed in nuclei and its localization was not detectably affected by EPS-evoked contractile activity (Fig. 4A, *upper two panels*). Unlike healthy control myotubes, some of the sIBM myotubes exhibited, especially after EPS-dependent contraction, obvious TDP-43 accumulations in cytoplasm (*lower three panels*, magnified image of white box is shown in the bottom panel), though such accumulation of TDP-43 was not evident in sIBM myotubes without EPS treatment. It should be noted that nuclear TDP-43 localization was also observed in 3T3L1 fibroblasts. In this set of experiments, we further examined a total of 6 individual human myotubes originating from 3 sIBM patients and 3 healthy control subjects and evaluated the TDP-43 deposits by counting numbers of myotubes possessing intracellular TDP-43 accumulations. All three healthy control myotubes only rarely displayed TDP-43 deposits, regardless of EPS-evoked contraction. In contrast, some populations of sIBM myotubes had TDP-43 deposits after EPS-evoked contraction, especially those derived from sIBM patients #4 and #6, while there were fewer deposits in those from patient **#**5 (Supplementary Information, Fig. S2). Summarizing the data demonstrated significant increases in numbers of myotubes displaying cytoplasmic TDP-43 accumulation only in the sIBM group after EPS treatment (Fig. 4B). In addition, within the sIBM myotubes displaying cytoplasmic TDP-43 deposits, their localization in the nuclei appeared to be relatively low^21^. Indeed, there were significantly fewer TDP-43-positive nuclei in sIBM myotubes after EPS treatment (Fig. 4C). sIBM and healthy control myotubes expressed similar levels of TDP-43 mRNA, which were not altered by EPS treatment (Fig. 4D).

In contrast to EPS-induced alterations in TDP-43 localization in sIBM myotubes, the p62 localization pattern was not influenced by EPS-evoked contraction in any of the 6 myotubes studied, i.e., regardless of whether they were derived from sIBM patients (Fig. 5A, *lower three panels*) or healthy control subjects (Fig. 5A, *upper two panels*), and the total p62-positive areas in myotubes also did not differ among the experimental conditions (Fig. 5B). The p62 mRNA levels were similar in the sIBM and healthy control myotubes, while showing slight but significant upregulation in response to EPS-evoked contractile activity (Fig. 5C). As noted above, both sIBM and healthy control myotubes all exhibited similar contractile ability upon EPS treatment in the feeder-supported in vitro exercise model, and no obvious impairment in contractile activity was detected in any of the 3 myotube preparations from individual sIBM patients, at least under the present experimental conditions (1 Hz, 4-ms, 20 V/25 mm for total 24 h) (*data not shown*).

## Discussion

Contractile activity is a fundamental function of skeletal muscles, which obviously contributes not only to the maintenance of healthy muscle properties but may also trigger various forms of muscle damage, depending on the loading intensity and modality, that may on occasion be related to the pathogenic processes of muscular diseases^22^. In the present study, we succeeded in establishing a “feeder-supported in vitro exercise model” using human-origin muscle cells that readily allows highly-developed contractile human myotubes to be obtained, with manipulatable contractility in response to the application of EPS treatment (Figs. 1, 2). By employing a murine fibroblast cell line, i.e., cells from a different species, as the feeder, biological responses upon EPS-evoked contraction originating only from the human myotubes could be selectively and accurately evaluated with species-specific RT-PCR analysis (Figs. 3, 4, 5).

The feeder-cell system has been widely used for various cell types^23^. Several studies have, in fact, demonstrated the supportive actions of a feeder layer of fibroblasts on myotube differentiation, functions and their survival^24–26^, though fibroblasts may also have a slight adverse effect on myotube formation^27^. In the present study, after a sufficient period for myotube formation on the 3T3L1 fibroblast feeders, we meticulously showed that mouse 3T3L1 fibroblasts do not fuse into human-origin multinucleated myotubes as assessed by immunofluorescent staining (Fig. 2A) and found that the human myotubes were highly endowed with contractile ability resulting in myokine upregulations upon EPS treatments, regardless of whether the samples had been obtained from sIBM sufferers or normal controls (Figs. 2, 3, 4, 5). Given the crucial involvement of substratum stiffness in cellular functions^28,29^, the feeder layer of fibroblasts apparently provides a favorable elastic substratum augmenting the development of contractility in human myotubes, overcoming the problem, even with appropriate EPS treatment, of endowing them with contractility in a culture system^7^.

Applying this feeder-supported in vitro model for myotubes originating from primary human satellite cells obtained from sIBM patients, we obtained evidence that sIBM myotubes basically possess full contractile capability, similar to that of healthy control myotubes in terms of contractility development and the associated vigorous activity (Fig. 3). Intriguingly, however, immunofluorescent analysis revealed that subcellular localization of TDP-43, but not of p62, differed in some of the sIBM myotubes especially after EPS-evoked contraction, a finding not observed in the healthy control myotubes (Figs. 4, 5). Thus, the feeder-supported in vitro exercise model established in the present study has the potential to serve as a useful EPS-based co-culture system applicable to investigating not only physiological but also pathophysiological consequences of actual contractile activity of muscle cells in vitro. Although caution must certainly be exercised when interpreting the data obtained, due to the co-existence of the feeder fibroblasts, this advantage may allow us to elucidate details of the intrinsic nature of a patient’s muscle cells, especially in response to an actual contractile burden, which could not be placed on bedridden patients suffering from severe muscular diseases such as sIBM.

At present the underlying mechanisms and their possible pathogenic consequences, whereby EPS-evoked contractile activity results in the formation of cytoplasmic TDP-43 accumulation only in some of sIBM myotubes, remain unclear. Normally, TDP-43 is predominantly localized within the nucleus^30^. However, abnormal extranuclear TDP-43 inclusions, such as morphologically granulo-filamentous or amorphous structures, have been recognized as one of the most prominent features of sIBM myofibers^21,31,32^. Indeed, immunostaining analysis of biopsy samples confirmed all three of our sIBM patients to have myofibers possessing pTDP-43-positive and p62-positive foci, though there were far fewer pTDP-43-positive than p62-positive myofibers (Fig. S1), as previously reported^19,21^. Thus, our observations in the sIBM myotubes indicate that the EPS-evoked contraction-inducible cytoplasmic TDP-43 accumulation detectable in some of sIBM myotubes may well be related to sIBM pathology. In sIBM patients, the occurrence of such sarcoplasmic TDP-43 aggregation reportedly varied, among studies, from ~ 23 to ~ 77%^19,21,33^. We detected the cytoplasmic TDP-43 accumulations in response to EPS treatment in about 30% of myotubes in two of the three distinct samples from sIBM myotubes but not in myotubes from any of the healthy subjects (Fig. 4B, Fig. S2). Moreover, nuclear TDP-43 extrusion increased significantly in sIBM myotubes in response to EPS treatment (Fig. 4C). While TDP-43 mRNA levels in sIBM were reportedly increased in some studies^33^ but not in others^34^, expression levels of TDP-43 in healthy and sIBM myotubes grown on fibroblast feeders were similar and were not influenced by EPS-evoked contractile activity, at least during the 24 h period of EPS application (Fig. 4D). Thus, we can reasonably speculate that EPS-evoked contraction-inducible cytoplasmic TDP-43 accumulation is chiefly achieved by translocation of TDP-43 to cytoplasmic regions from nuclei in the sIBM myotubes under the in vitro experimental condition.

Taking our present in vitro data and the aforementioned clinical in vivo data together, abnormal extracellular TDP-43 accumulations are indeed sporadic and such TDP-43 abnormalities might occasionally be induced even under cell culture conditions wherein there are no other cell types such as immune cells that may directly and/or indirectly be related to the pathogenesis of sIBM^35,36^. In an attempt to elucidate possible pathogenic consequences related to abnormal cytosolic TDP-43 accumulation in sIBM myotubes, we examined expression levels of sortilin mRNA splicing variants including exo17b which is reportedly affected by TDP-43 abnormalities^37,38^, but identified no alterations in expression profiles between sIBM and healthy control myotubes regardless of whether or not EPS treatment was applied (*data not shown*). It should be also noted that all three sIBM patients similarly exhibited the typical sIBM phenotype of rimmed vacuoles. Thus, future studies are warranted to elucidate potential pathogenic contributions of abnormal TDP-43 accumulation in sIBM myotubes as well as to clarify the mechanism underlying the abnormal TDP-43 accumulation triggered by EPS-evoked contraction in some, but not all, sIBM myotubes. Nevertheless, our data strongly suggest the existence of intrinsic vulnerability, affecting the sIBM myotube itself, that emerges only upon actual contractile activity but not under routine culture conditions^17^.

p62 serves as a cargo receptor in the degradation of ubiquitinated proteins and organelles during autophagic responses^39,40^ and abnormal p62 accumulation has thus been implicated in the pathogenesis of sIBM^12,41^, due to a mechanism involving impaired autophagic clearance of aggregation-prone proteins including amyloid precursor protein^42,43^. However, unlike the very distinctly different TDP-43 localizations in sIBM and healthy control myotubes, the p62-positive small granule-like (puncta) structures were similar, being scattered throughout the cytoplasm in both sIBM and healthy control myotubes regardless of whether or not of EPS treatment had been applied (Fig. 5A,B). In both healthy and sIBM myotubes after EPS-evoked contraction, the p62 mRNA level was slightly but significantly upregulated (Fig. 5C), though these subtle changes were not detectable by immunofluorescent analysis. In the present study, we used DMEM enriched with all amino acids to enhance contractile development^2^. Since autophagy is generally induced under nutrition-deficient (starved) culture conditions^44,45^, we can only speculate that our culture conditions during EPS treatment likely influenced the autophagic responses in which p62 might participate. In addition, we examined only one EPS treatment condition because this EPS protocol consistently provides highly reliable and reproducible outcomes from in vitro exercise models^2,4,7^. Nonetheless, the current results do not allow us to assert the significance of the aforementioned TDP-43 abnormality observed in some sIBM myotubes in this study. Moreover, the underlying mechanism of abnormal TDP-43 accumulation triggered by EPS-evoked contraction in some, but not all, sIBM myotubes, remains unclear. These crucial issues thus need to be addressed in future studies by examining large numbers of myotubes originated from sIBM patients. As the limitations of this study, it should also be noted that control and sIBM samples were taken from different muscle tissues, i.e., the biceps brachii or rectus femoris muscles from two male and one female sIBM patient, all 69–86 years of age, while the subscapularis muscles were sampled in three healthy male subjects 60–68 years of age. Thus, while their ages are relatively well matched, with all being categorized as elderly, background features, i.e., sex and tissue type differences, might have influenced the phenotypes of myotubes observed in the present study. This issue needs to be carefully evaluated in a future study. Accordingly, further improvements of the feeder-supported in vitro exercise model, aimed at appropriately mimicking the pathogenic processes of myopathic diseases, are essential. Prospectively eliciting such processes in response to a contractile burden under certain experimental culture conditions such as various nutritional states, different exercise intensities/modalities, in the presence of other cell types, and so on, would likely provide valuable clinical and research insights.

In the present study, we focused on contractility development in human-origin myotubes, and its impacts, in response to actual contraction, on intrinsic muscle properties. For sIBM myotubes particularly, this co-culture system may also be useful for elucidating higher-order functional interplays between contracting muscle cells and surrounding fibroblastic cells^25,27^ as it can regenerate and mimic muscle tissue-like environments that contain numerous fibroblastic cells. In this case, co-culture with fibroblastic cells from the same individual with sIBM, instead of mouse fibroblasts, as a feeder, may establish situations more pathologically similar to the disease state by allowing cells to communicate with each other as observed in the samples derived from patient muscle biopsy specimens.

In summary, we established a “feeder-supported in vitro exercise model” applicable to sIBM myotubes derived from the primary satellite cells of sIBM patients. This newly established “feeder-supported in vitro exercise model” enables us to readily produce contractile human-origin myotubes that can be subjected to further experimental and diagnostic analyses to gain details regarding exercise-evoked biological responses in vitro. Thus, our approach, using feeder cells, further expands the usefulness of the “in vitro exercise model”.

## Methods

### Materials

Dulbecco’s modified Eagle’s medium (DMEM), and Ham’s F-10 medium were purchased from Fujifilm Wako Pure Chemical Corp. (Osaka, Japan). Penicillin/streptomycin, and trypsin–EDTA were obtained from Thermo Fischer Scientific (Rochester, NY, USA). Cell culture equipment and rectangular 8-well plates were obtained from BD Biosciences (San Jose, CA, USA) and Thermo Fisher Scientific, respectively. SUMILON Cell-disk LF1 was from Sumitomo Bakelite Co. LTD (Tokyo, Japan). Calf serum (CS) and fetal bovine serum (FBS) were obtained from BioWest (Nuaille, France). Matrigel was obtained from Corning (#354230, NY, USA). Unless otherwise noted, all chemicals were of the purest grade available from Sigma Chemical (St Louis, MO, USA) or Fujifilm Wako Pure Chemical Corp.

### Cell culture

Human skeletal muscle myoblasts (HSMM) (Cat.# CC-2580) were purchased from Lonza (Walkersville, MD, USA) and were cultured following the vendor’s instructions. Primary human satellite cells were obtained by fluorescent-activated cell sorting (FACS) from muscle biopsy tissues at Tohoku University Hospital under the approval of the Ethics Committee of Tohoku University, and written informed consent was obtained from all subjects (see below). The primary human satellite cells were amplified and stocked in liquid N_2_ as previously reported^46^. Primary satellite cells were cultured in a growth medium containing DMEM/Ham’s F10 mixture supplemented with 20% FBS, 1% penicillin–streptomycin, 1% chicken embryonic extract (United States Biological, Salem, MA, USA), and 2.5-ng/ml basic fibroblast growth factor (Thermo Fischer Scientific, Waltham, MA, USA) at 37 °C under a 5% CO2 atmosphere. Mouse 3T3L1 fibroblasts were maintained in DMEM containing 4.5 g/l glucose supplemented with 10% CS, 4 mM l-glutamine, 30 μg/ml penicillin, and 100 μg/ml streptomycin (growth medium) at 37 °C under a 5% CO2 atmosphere.

For the feeder layer, 3T3L1 fibroblasts were seeded at a density of 0.5 × 10^5^ cells onto an 8-well plate. Three hours after seeding, human myoblasts (activated satellite cells) were seeded onto the feeder layer of 3T3L1 fibroblasts at a density of 3 × 10^5^ cells in each well of an 8-well plate in 3 ml of growth medium. For immunofluorescent analysis, 3T3L1 fibroblasts were seeded onto a Cell-Disc LF in a 24-well plate at a density of 1 × 10^4^ cells. Three hours after seeding, human myoblasts (activated satellite cells) were seeded onto the feeder 3T3L1 fibroblasts on the Cell-Disc LF in 1 ml of growth medium at a density of ~ 6 × 10^4^ cells. Two days after plating, differentiation was induced by switching the growth medium to DMEM supplemented with 5% horse serum, 30 μg/ml penicillin, and 100 μg/ml streptomycin (differentiation medium). The differentiation medium was changed every 24–48 h during the 7–8 days of differentiation.

### Electrical pulse stimulation (EPS)

The differentiated human myotubes on feeder 3T3L1 fibroblasts in 8-well plates or on Cell-Disc LF being transferred into an 8-well plate, were placed in a chamber for EPS (C-Dish; IonOptix, Milton, MA). EPS (1 Hz, 4-ms, 20 V/25 mm) was applied to the cells in the C-Dish using a C-Pace 100 pulse generator (IonOptix). DMEM containing 2% CS supplemented with 200% amino acids (Sigma) was used during the EPS treatments, as previously reported^2^. In the present study, 24 h in total of EPS (8 h × 3 times with 1 h intervals between sessions) were applied and the cells were then harvested for analyses.

### Calculation of movement index

The index of movement was calculated by the differential image subtraction method with a slight modification, as we reported previously^1,7^. Briefly, high-quality images of cells were taken sequentially with a CCD camera (Hamamatsu C3077-70, Japan) and a dissection microscope (Olympus CKX41) equipped with a 20× objective lens during EPS. We obtained the movies at completion (during the final ~ 15 min of stimulation) of the total 24 h of EPS treatment applied for the contractile activity evaluations. The differential images were obtained by subtracting the relaxation image from the contraction image. Given that the difference in pixel intensity between the first (contraction) and successive (relaxation) images is due to changes in the movement of scattering objects, the overlaid images of these subtracted images indicate moving parts. The calculated average intensity of the differential image indicates the amount of myotube movement (movement index). Three different fields under each culture condition were used for evaluation of the movement index.

### Immunofluorescence analysis

After the experimental treatments, cells were washed with PBS and fixed for 20 min with 2% paraformaldehyde in PBS containing 0.1% Triton X-100, then washed and blocked in PBS containing 5% CS and 1% BSA at room temperature. For immunofluorescence analysis, we used anti-human nuclear antigen (HNA) antibody (MAB4470, R&D Systems, Minneapolis, MN, USA), anti-caveolin 3 antibody (PA1-066, Affinity BioReagents, CO, USA), anti-sarcomeric α-actinin (#A7811, Sigma), anti-TDP-43 (#10782-2, Proteintech, Rosemont, IL, USA), and anti-p62/SQSTM1 (#18420-1, Proteintech) as the first antibodies, and Alexa Fluor 488-conjugated anti-mouse IgG and Alexa Flour 594-conjugated anti-rabbit IgG as the secondary antibodies (Thermo Fischer Scientific) at 1:100 and 1:1000 dilutions, respectively, in a solution of 1% BSA in PBS. The samples were mounted on glass slides with Vectashield (Vector Laboratories, Burlingame, CA, USA) and observed with a confocal fluorescence microscope (Fluoview FV-1000; Olympus, Tokyo, Japan) with an oil-immersion objective lens (PLANPON60xOSC2, NA 1.4, Olympus) and ASW v.1.3 software (Olympus). The fluorescence of DAPI, Alexa 488, and Alexa 555 was excited at 405, 488, and 543 nm laser wavelengths and detected through BA430-470, BA505-525, and BA560-600 nm bandpass filters, respectively. The pinhole diameters are set automatically according to the selected dyeing method, and imaging was performed at 8.0–10 μs/pixel (Scan speed) for 1024 × 768 (4:3) or 1024 × 1024 (1:1) pixels applying a sequential scan mode with Kalman filtering. The number of myotubes displaying cytoplasmic TDP-43 aggregations and the total number of myotubes, as assessed by the sarcomeric α-actinin staining profile for discriminating feeder 3T3L1 fibroblasts, were counted in each field, and the ratio of aggregated TDP-43-positive myotubes vs. the total number of myotubes was evaluated. Images were imported into Adobe Photoshop 6.0 (Adobe Systems, San Jose, CA, USA) for processing. Three independent experiments were performed under each condition.

### Quantification of TDP-43-positive nuclei, intracellular TDP-43 accumulation, and p62 puncta

For evaluating TDP-43-positive nuclei and intracellular TDP-43 accumulations, we used Fiji ImageJ software (NIH, Bethesda, MD, USA). Briefly, we first defined the myotube boundary according to the anti-sarcomeric-α-actinin immunofluorescent signal, and then obtained all the nuclei (by DAPI) and TDP-43-positive nuclei according to their respective immunofluorescent signals. The number of all nuclei in the myotube was used as the denominator, and the number of all TDP-43-positive nuclei was used as the numerator to obtain the ratio of TDP-43-positive nuclei in the myotube. For evaluating cytoplasmic TDP-43 accumulation, we used the Image calculator function of Fiji ImageJ software. Briefly, we first determined the entire TDP-43-positive area and the TDP-43-positive nuclear area of the myotubes by delineating sarcomeric-α-actinin staining and DAPI staining, respectively. Then, we subtracted the TDP-positive nuclear area from the total TDP-43 area to obtain the fluorescent intensity of the TDP-43 accumulated in cytoplasmic regions. Finally, we counted numbers of total myotubes and the myotubes exhibiting cytoplasmic TDP-43 accumulation to determine the ratio of cytoplasmic TDP-43-positive myotubes. For the p62 area, we calculated the p62-positive puncta area to the total myotube area using Fiji ImageJ software.

### Quantitative real-time PCR (qRT-PCR) analysis

Total RNA was extracted from cells employing TRI reagent (Molecular Research Center Inc., Cincinnati, OH, USA) and cDNA was then synthesized using a Transcriptor First Strand cDNA synthesis kit with oligo-dT primers (Roche, Basel, Switzerland). Next, qRT-PCR was performed with SsoAdvanced Universal SYBR Green Supermix, and detected with a Bio-Rad CFX Connect Systems (Bio-Rad Laboratories Inc., Hercules, CA, USA). The relative expression levels of the target genes were calculated using the 2^−∆CT^ method with reference genes. The following primers for qRT-PCR analyses were employed; for human CXCL1, 5ʹ-GCT TGC CTC AAT CCT GCA TC-3ʹ and 5ʹ-GGT CAG TTG GAT TTG TCA CTG T-3ʹ; for human IL6, 5ʹ-ATC TGG ATT CAA TGA GGA GAC T-3ʹ and 5ʹ-TGT TCC TCA CTA CTC TCA AAT CTG-3ʹ; for human TDP-43, 5ʹ-GAG AAG TTC TTA TGG TGC AGG TC-3ʹ; 5ʹ-GCT CAT CTT GGC TTT GCT TAG-3ʹ; for human p62/SQSTM1, 5ʹ-GCG TAG AAT TGC AGG TCT CTG T-3ʹ and 5ʹ-TCA CTT GTT TTG CTG CCC TAA ATG-3ʹ; for human RPLP0, 5ʹ-GGA AAC TCT GCA TTC TCG CT-3ʹ and 5ʹ-GCA AGT GGG AAG GTG TAA TCC-3ʹ.

### Patients

The three patients, who had been clinically diagnosed with sIBM at Tohoku University Hospital, ranged in age from 69 to 86 years and had no family history of muscle diseases. The basis of the sIBM diagnosis was the presence of rimmed vacuoles in muscle biopsy tissue. The distribution of muscle weakness, creatin kinase activity, and the results of electrophysiological studies were evaluated during the disease course. Clinical data obtained from all sIBM patients, two males and one female, who had been hospitalized between 2015 and 2016 in the Department of Neurology of Tohoku University hospital, were evaluated^15^. As the healthy controls, we used intact subscapularis muscle specimens from three male patients, ranging in age from 60 to 68 years, who had agreed to undergo biopsy during surgical repair of arthroscopic rotator cuff tears^7,46^.

### Immunostaining

Immunohistochemical staining was carried out using the avidin–biotin complex immunostaining method as previously described^47^, with mouse monoclonal antibodies (diluted to 1:200) raised against p62 (M162-3, MBL, Tokyo, Japan) and mouse monoclonal antibodies (diluted to 1:5,000) raised against phosphor-TDP-43 (Clone 11–9, pS409/410, Cosmo Bio, Tokyo, Japan).

### Human satellite cell isolation and proliferation

Human satellite cells of both the three sIBM patients and the three healthy subjects were isolated from muscle tissue biopsy specimens, as previously reported^15,46^. This study was approved by the Tohoku University Hospital Institutional Review Board (approval numbers: 2014-1-703 and 2019-1-493), and written informed consent was obtained from all participants. All experiments were performed in accordance with relevant guidelines and regulations.

Briefly, the muscle tissue was minced and digested with collagenase, filtered through a 70-μm cell strainer (BD Biosciences, Franklin Lakes, NJ, USA) and then subjected to immunostaining for FACS. Cells were incubated with an Fc receptor blocking solution and then labeled with fluorescein isothiocyanate (FITC)-conjugated anti-CD45 (clone HI30), FITC-conjugated anti-CD11b (clone ICRF444), FITC-conjugated anti-CD31 (clone WM59), phycoerythrin (PE)/Cy7-conjugated anti-CD34 (clone 581), allophycocyanin (APC)-conjugated anti-CD56 (clone MEM-188), and PE-conjugated anti-PDGFR*α* (clone 16A1). The negative set included blood markers CD11b and CD45, and endothelial markers CD31 and CD34. While CD34 is known to be expressed by the majority of mouse satellite cells^48^, human muscle-derived CD34^+^ cells are myogenic and adipogenic, whereas CD34^−^ cells are myogenic but not adipogenic^49^. We therefore used CD34 as a negative selection marker. Human satellite cells were defined as single live mononuclear CD11b^−^CD31^−^CD34^−^CD45^−^CD56^+^ cells. FACS was performed on a FACS ARIA II flow cytometer (BD Biosciences). Cells were cultured in a growth medium and cultured at 37 °C in a 5% CO_2_ atmosphere. When cells reached 60–80% confluence, adherent cells were split onto a new Matrigel-coated 15-cm dish to expand the activated satellite cells. Activated satellite cells (myoblasts) were suspended in Cell Banker (TAKARA, CB011, Japan) and stored in liquid nitrogen.

### Statistical analysis

Statistical analyses were performed using Student’s *t*-test or ANOVA with Tukey’s multiple comparison test, and *p* values < 0.05 were considered to indicate a statistically significant difference unless otherwise specified. Data are expressed as means ± SE unless otherwise specified.

## Supplementary Information

Supplementary Figures.

Supplementary Video 1.

Supplementary Video 2.
