# Supplementary material for: Feeder-supported in vitro exercise model using human satellite cells from patients with sporadic inclusion body myositis
Source: Sci Rep. 2022 Jan 20;12:1082. doi: 10.1038/s41598-022-05029-w (PMC8776910; doi:10.1038/s41598-022-05029-w)
Supplement: Supplementary file 1 — Supplementary Figures. [file 41598_2022_5029_MOESM1_ESM.pdf]

Supplemental Information

Feeder-supported in vitro exercise model using human satellite cells from patients with sporadic inclusion body myositis

Yuqing Li, Weijian Chen, Kazumi Ogawa, Masashi Koide, Tadahisa Takahashi, Yoshihiro Hagiwara, Eiji Itoi, Toshimi Aizawa, Masahiro Tsuchiya, Rumiko Izumi, Naoki Suzuki, Masashi Aoki and Makoto Kanzaki

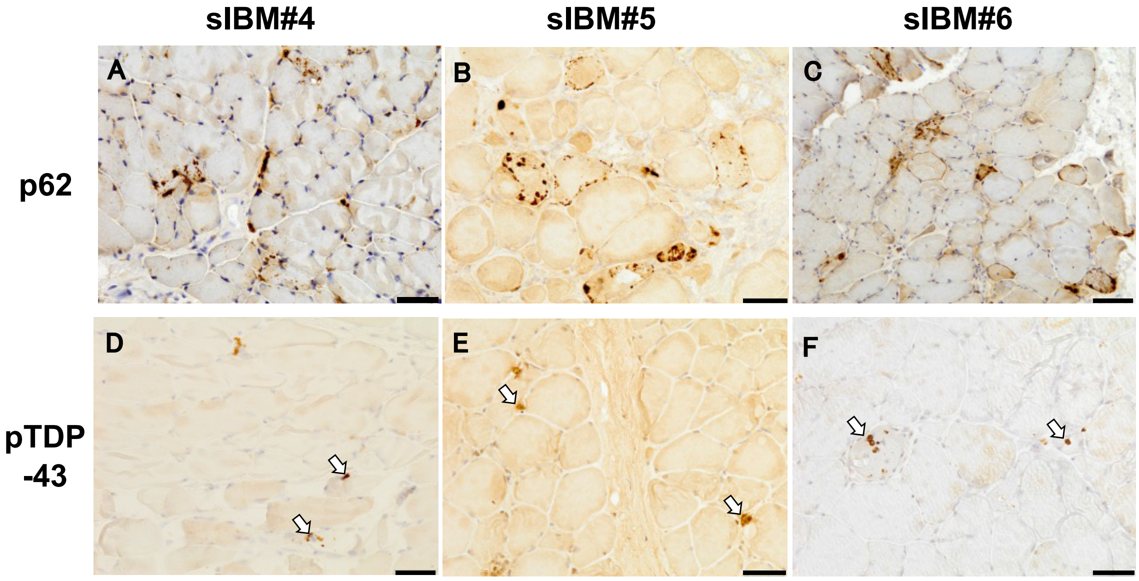

Figure S1. Immunohistochemical analysis of p62 (A-C) and TDP-43 (D-F) in three sIBM cases

Immunohistochemical staining was performed as described in Methods. Note scattered p62-positive myofibers (A-C) and phospho-TDP-43 positive myofibers (arrows in D-F). Scale bars = 50 µm (A-B, D-F) and 100µm (C).

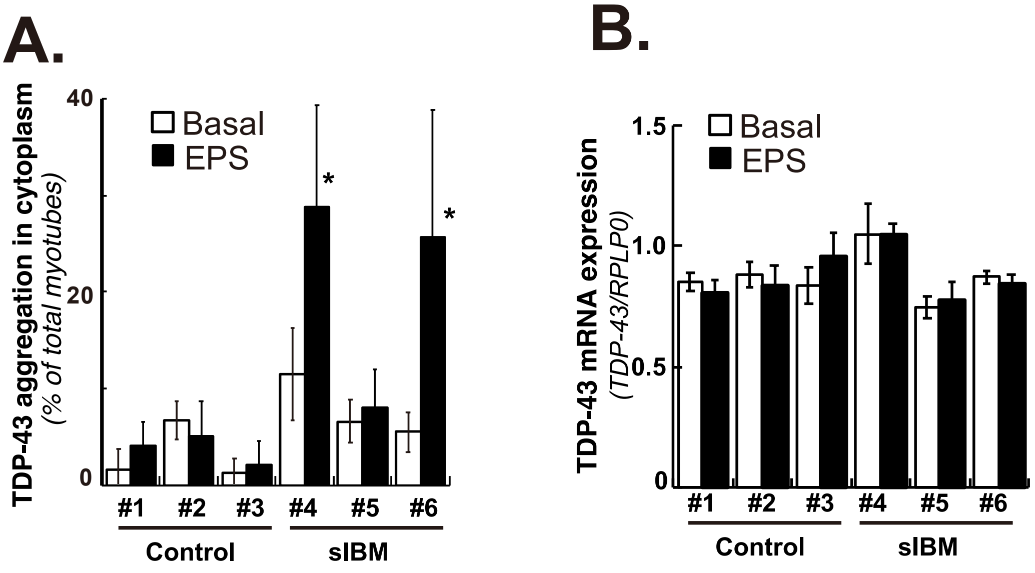

Figure S2. Cytoplasmic TDP-43 accumulation (A) and TDP-43 mRNA expression in myotubes from each of the subjects (related to Figure 4)

(A) Quantification of the ratio of myotubes displaying cytoplasmic TDP-43 accumulations in the myotube was determined from 40-75 myotubes in three independent experiments as described in the legend for Figure 4. The statistical significance of differences was analyzed using the student t-test; * $p < 0.05$, indicating the effects of EPS treatment.

(B) Total RNA was extracted and mRNAs for human TDP-43 and RPLP0 were evaluated by real-time PCR analysis. Data were normalized using human RPLP0 transcripts. This graph shows the results of three independent experiments performed using the same cell stock samples.
